# Development of a CMOS-Compatible Carbon Nanotube Array Transfer Method

**DOI:** 10.3390/mi12010095

**Published:** 2021-01-18

**Authors:** Chun Fei Siah, Lucas Yu Xiang Lum, Jianxiong Wang, Simon Chun Kiat Goh, Chong Wei Tan, Liangxing Hu, Philippe Coquet, Hong Li, Chuan Seng Tan, Beng Kang Tay

**Affiliations:** 1 Centre for Micro- and Nano-Electronics (CMNE), School of Electrical and Electronic Engineering, Nanyang Technological University, 50 Nanyang Ave, Singapore 639798, Singapore; chunfei001@e.ntu.edu.sg (C.F.S.); e190013@ntu.edu.sg (L.Y.X.L.); ChongWei@ntu.edu.sg (C.W.T.); lxhu@ntu.edu.sg (L.H.); TanCS@ntu.edu.sg (C.S.T.); 2 CNRS-NTU-THALES Research Alliances/UMI 3288, Research Techno Plaza, 50 Nanyang Ave, Border X Block, Level 6, Singapore 637553, Singapore; jxwang@ntu.edu.sg (J.W.); simon.goh@ntu.edu.sg (S.C.K.G.); Philippe.Coquet@cnrs.fr (P.C.); ehongli@ntu.edu.sg (H.L.); 3 Institut d’Electronique, de Micro Electronique et de Nanotechnologie (IEMN), CNRS UMR 8520-Université de Lille, 59650 Villeneuve d’Ascq, France; 4 School of Mechanical and Aerospace Engineering, Nanyang Technological University, 50 Nanyang Ave, Singapore 639798, Singapore

**Keywords:** carbon nanotubes, microelectronics fabrication, bonding, temperature

## Abstract

Carbon nanotubes (CNTs) have, over the years, been used in research as a promising material in electronics as a thermal interface material and as interconnects amongst other applications. However, there exist several issues preventing the widespread integration of CNTs onto device applications, e.g., high growth temperature and interfacial resistance. To overcome these issues, a complementary metal oxide semiconductor (CMOS)-compatible CNT array transfer method that electrically connects the CNT arrays to target device substrates was developed. The method separates the CNT growth and preparation steps from the target substrate. Utilizing an alignment tool with the capabilities of thermocompression enables a highly accurate transfer of CNT arrays onto designated areas with desired patterns. With this transfer process as a starting point, improvement pointers are also discussed in this paper to further improve the quality of the transferred CNTs.

## 1. Introduction

In the past few decades, carbon nanotubes (CNTs) have stimulated a large interest in the research world for their material properties. The discovery of their extraordinary mechanical [1], electrical [2], and thermal [3] properties prophesized CNTs as the next revolutionary material in numerous applications such as interconnect [4], thermal management [5], field-emission [6], and on-board electromagnetic shielding [7].

The integration of CNTs into real-world applications is faced with several difficulties [8]. First, the high growth temperature of CNTs, over 600 °C, is not compatible with complementary metal oxide semiconductor (CMOS) device fabrication processes. Second, a diffusion barrier layer [9] between the catalyst and the growth substrate is required for the growth of CNTs using chemical vapor deposition (CVD). This leads to an additional interfacial resistance between the substrate and CNTs. Third, the CVD processes deposit by-products such as amorphous carbon (a–c) [10] from unconsumed process gases, which can short-circuit the metal electrodes present on the surface of the substrate, reducing yield and device performance. Fourth, micro-fabrication processes such as photolithography and chemical-mechanical polishing can also affect the quality of CNT growth [11]. The non-planar and complex geometries of fabricated microelectronic device substrates can also limit the quality of CNT growth. For example, a catalyst layer located deep inside a high-aspect ratio via can be starved off the required carbon precursors for proper CNT growth. Lastly, in a CVD process, vertically-aligned CNTs (VACNTs) can only be deposited on a selected choice of substrates, typically SiO_2_/Si wafers or copper substrates that can allow for a high density of iron oxide clusters as nucleation sites for the growth of CNTs [12]. The chosen substrates must be stable to prevent the interaction and poisoning of the catalyst layer during CNT growth.

In this article, a method for the transfer of VACNTs is achieved by utilizing a solder-bonding technique through flip-chip bonding. VACNT-based devices fabricated using the transfer method have the advantage of improved physical and electrical contact at CMOS-compatible temperatures without the need for complex pre-treatment of the target substrates. As the target substrate is excused from the CNT growth process, it does not undergo high fabrication temperatures and is not exposed to the a–c deposits that may compromise its reliability. This paper details the transfer method of VACNT arrays to target device substrates, followed by a discussion of the results and possible areas that can further improve the yield and quality of the VACNT transfer method.

## 2. Materials and Methods

### 2.1. Preparation of Carbon Nanotube on Donor Substrate

**Figure 1:**
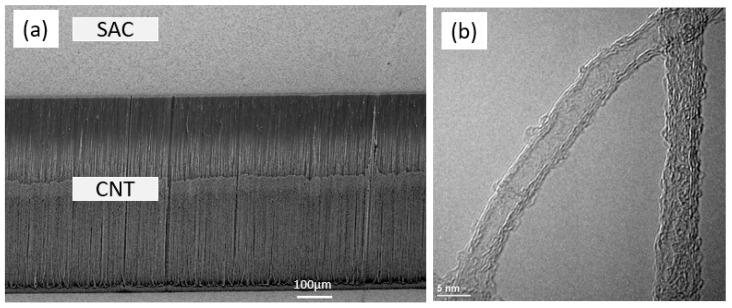
(**a**) Silver-copper alloy (SAC) deposited on vertically-aligned carbon nanotube (VACNT) does not affect the alignment of the array; (**b**) Transmission electron microscopy (TEM) image of as-grown CNT.

The catalyst layer for CNT growth was first prepared on a donor silicon substrate by depositing a 10 nm aluminum oxide (Al_2_O_3_) as the diffusion barrier layer, followed by a 1 nm iron (Fe) catalyst layer by a physical vapor deposition (PVD) system. The Al_2_O_3_/Fe catalytic substrates were then placed into a commercial CNT growth system where acetylene is used as the carbon precursor at a growth temperature of 675 °C. CNT growth height was controlled by varying the growth time. After growth, the donor substrates were cooled down to room temperature before unloading. Transmission electron microscopy (TEM) characterization on the as-grown CNTs show multi-walled CNTs with an average diameter of 5 nm and an average number of five walls. Next, a layer of 35 nm titanium (Ti) followed by 1 µm of tin-silver-copper alloy (SAC) was deposited using PVD with a patterned hard mask onto the as-grown CNTs to act as the binding material, where Ti serves as an adhesion layer between the CNTs and the SAC layer. As shown in Figure 1a, the deposited metal layers do not affect the vertical alignment of CNT.

### 2.2. Preparation of Target Substrate

In order to bond the SAC-deposited CNTs to another substrate, a target substrate was prepared using ultra-violet (UV) photolithography to pattern the substrate before coating a 35 nm Ti/1 µm gold (Au) layer to define the desired regions of transfer. This metallization was chosen as it is a common material for electrodes whilst being resistant to oxidation at the process temperatures (<300 °C). The target substrate used is a polished Si wafer with a smooth surface (average roughness < 5 nm).

### 2.3. Transfer of Carbon Nanotubes

**Figure 2:**
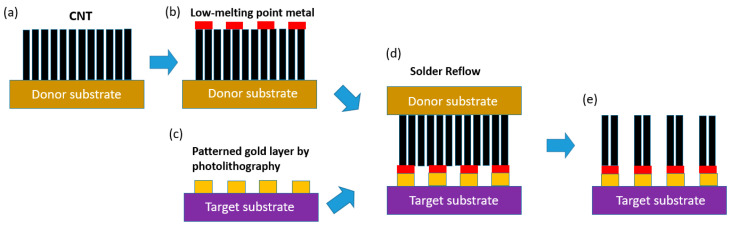
Schematic of CNT transfer method: (**a**) vertically-aligned CNT growth; (**b**) evaporation of low-melting point metal such as solder through hard mask onto CNT array; (**c**) preparation of target substrate; (**d**) solder reflow process to bond the CNT array and target substrate; (**e**) separation of CNT array from donor substrate.

To transfer CNTs from the donor substrate to the target substrate, the CNTs need to be bonded to the target substrate. This is possible by placing the SAC-coated CNTs and Au-coated surface in contact before performing a solder reflow step as described in [13] at temperatures below 250 °C. The solder reflow process includes the steps: pre-heat; soaking; reflow; and cooling at designated temperatures. Each step needs to be carefully executed due to their impact on the wetting properties and microstructure of the solder, affecting the bonding quality. The solder reflow step is performed in the presence of an applied external load to ensure a good contact between CNTs and target substrate without air gaps. The transfer of CNTs onto their designated areas can be achieved with precision by using a flip-chip bonder. Finally, a separation process removes the donor substrate to achieve the final structure. This is done by applying forces on the donor and target substrate in opposite directions with a separation tool. A schematic of the CNT transfer method is prepared and shown in Figure 2.

## 3. Results and Discussion

**Figure 3:**
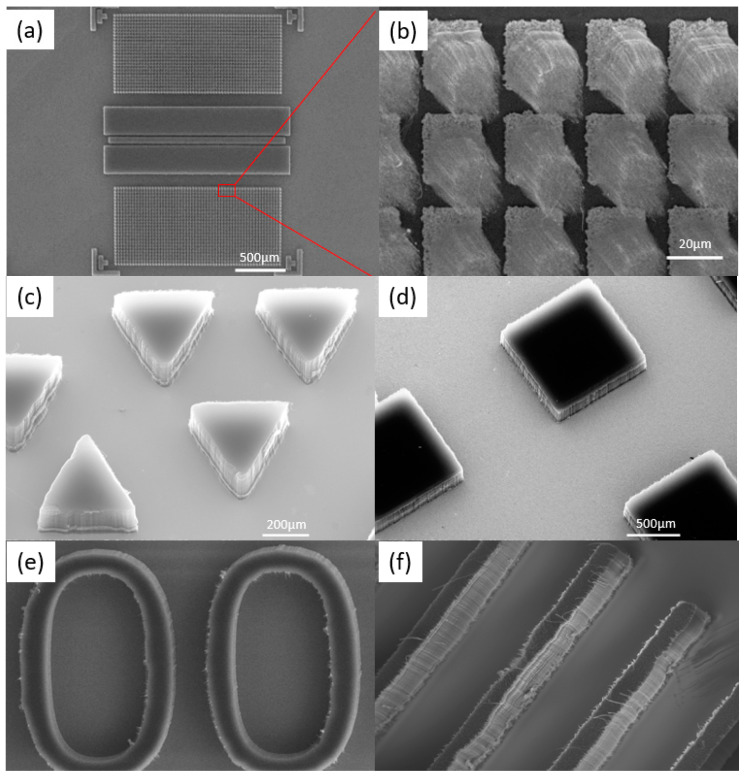
(**a**,**b**) Transferred CNT with bump size of 25 µm and a pitch of 10 µm with surrounding bumps; (**c**–**f**) multiple shapes of transferred CNT—for (**e**,**f**) the width of the CNTs patterns are 200 µm and the height of the CNT 500 µm.

CNTs were bonded to an as-prepared target substrate by using a flip-chip bonder. A high precision and conformability can be achieved with the transfer onto the designated areas on the target substrates, as shown in Figure 3. In Figure 3a,b, the size of the transferred bumps is 25 µm with a pitch of 10 µm between surrounding bumps. Figure 3c–f show multiple shapes with sharp or rounded edges transferred with no change in the vertical alignment of CNTs. Since the target substrate is not exposed to CNT growth, it remains free of a–c deposits and continue to be so after the transfer process. No residue of SAC is visible on the target substrate, indicating a clean and controllable process.

**Figure 4:**
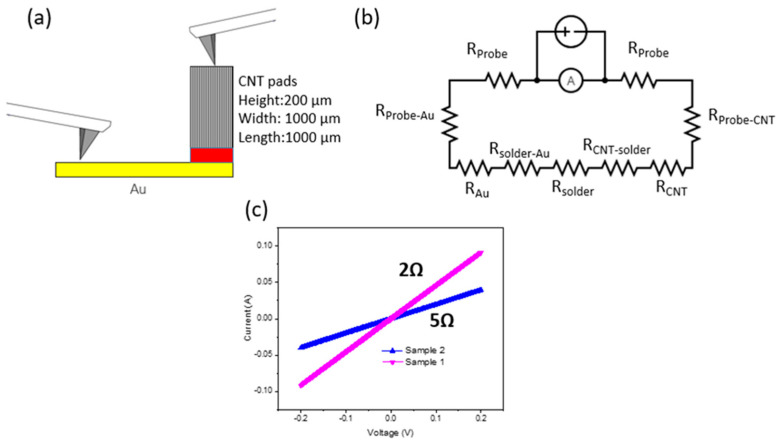
(**a**) Measurement setup to measure IV-response of transferred CNT array at given CNT dimension; (**b**) schematic of electrical connection in measurement setup; (**c**) measured IV-responses and corresponding resistances.

To characterize the electrical properties of the transferred CNTs, a current-voltage (IV) measurement was done on a 1 mm^2^ transferred CNT pad with a transferred height of 200 µm, to obtain the through-plane resistance of transferred CNTs. The measurement schematic with the structure is illustrated in Figure 4a. A voltage was applied between the two probes to measure the current that passed through the transferred CNT array, the CNT structure, followed by the Au-SAC interface and the gold electrode before terminating at the second probe.

IV-responses on two CNT pads with a voltage sweep from −0.2 to 0.2 V gives a linear relationship. This shows the ohmic relationship of the bonding interface. A breakdown on every possible element contributing to resistance has been presented in Figure 4b. As shown in Figure 4c, the calculated resistance ranges from 2 to 5 Ω. The resistances are still larger than pure metallic contacts and show room for improvement. Specifically, resistance in the contact can be improved by changing the binding material to one with a lower resistivity like the Sn-Au solder. If the Sn-Au solder is used, it has the benefit of a flux-less solder reflow process [14]. As the flux used in conventional solder reflow penetrates the CNT array when melted, causing densification and shape distortion. Another possible way to achieve a good electrical contact is by using the thermocompression bonding method. It has the advantage of reducing the material variety at the bonding interface. Thermocompression using an Au-Au interface [15] as the bonding interface between CNTs and the target substrate is achieved and intended to be used in thermal interface material (TIM) applications.

To improve conductivity of CNTs, the CNT array can also be treated with plasma prior to the deposition of the binding material to remove the closed caps of the CNTs, enabling the inner walls to contact the binding material [16] as well as improve the wettability of the CNTs to it [17]. The number of channels for electron conduction can be increased in this way and hence, the overall electrical conductivity of the transferred CNT array can be improved [18,19] to a limit of multichannel ballistic transport as referenced from a multitude of prior research. The measured conductance of multichannel ballistic transport is much greater than 2*G*_0_; its relation is shown in Equation (1). Where *G*_0_ is the total conductance when the length of a conductor is smaller than the electron mean free path and the electron transport is ballistic, *e* is the free electron charge and h is plank’s constant:

(1) $$G_{0}=\;\frac{2e^{2}}{h}.$$

By using the VACNT transfer method described above, the resultant joint between transferred CNTs and target substrate is CNT-Ti-SAC-Au. In comparison to a similar work by Liu et al. [20] that reported a CNT-Ti-Au-indium-Au contact, a reduction of one layer of binding material was achieved due to the higher hardness of SAC; the requirement of an additional Ti/Au reinforcement layer in the indium process is eliminated, whilst maintaining the transfer yield. The reduction in the number of metal junctions helps to decrease process varieties without negatively affecting the contact resistance. The use of SAC as the binding material also makes the transfer method a CMOS-compatible process as the reflow step is performed below 250 °C.

## 4. Conclusions

A CNT transfer method has been developed and shown to produce an ohmic electrical contact with a deposited gold layer, achieving a calculated resistance of <10 Ω. Furthermore, the transfer method separates the target substrate from CNT growth and hence, no a–c deposits can be found on the substrate. Using the transfer process as an example, a few pointers have been uncovered to improve the quality of transfer as well as the quality of the CNT structure, e.g., use of a binding material with lower resistances and plasma-treatment of the CNT arrays. By using the transfer method described above, the resultant joint is CNT-Ti-SAC-Au, which has lesser interfacial layers of binding material than reported in [20]. Since the SAC reflow step is performed below 250 °C, the transfer method developed using SAC is CMOS-compatible. Improvements in the CNT transfer technique will uncover opportunities in the commercial applications of CNTs, such as interconnects [4], thermal management [5], field-emission [6], and on-board electromagnetic shielding [7].

## 5. Patent

The work described above is patented [21].
